# Strain Echocardiography in MINOCA: Diagnostic and Follow-Up Implications

**DOI:** 10.3390/jcm15134934

**Published:** 2026-06-25

**Authors:** Mustafa Lutfi Yavuz, Pelin Karaca Ozer, Elif Ayduk Govdeli, Mehmet Aydoğan, Mehmet Akif Parlar, Samim Emet, Ali Elitok, Fehmi Mercanoglu

**Affiliations:** 1 Department of Cardiology, Faculty of Medicine, Istanbul University, 34093 Istanbul, Türkiye; pkaracaozer@gmail.com (P.K.O.); elifayduk@gmail.com (E.A.G.); dr.maydogan@hotmail.com (M.A.); samim03@hotmail.com (S.E.); alielitok@yahoo.com (A.E.); fmercanoglu@gmail.com (F.M.); 2 Department of Medical Oncology, Ankara Bilkent City Hospital, 06800 Ankara, Türkiye; m.akifparlar@gmail.com

**Keywords:** MINOCA, strain echocardiography, subclinical myocardial dysfunction

## Abstract

**Background/Objectives**: Myocardial infarction with non-obstructive coronary arteries (MINOCA) represents a heterogeneous clinical entity in which patients exhibit symptoms and biomarker evidence of myocardial infarction despite the absence of significant coronary stenosis. This study aimed to evaluate the diagnostic and follow-up value of left ventricular global longitudinal strain (LV-GLS) in patients with MINOCA and to investigate its association with inflammatory and glycemic biomarkers. **Methods**: A total of 287 patients presenting with acute coronary syndrome were prospectively enrolled. Based on coronary angiographic findings, patients were classified into two groups: myocardial infarction with obstructive coronary arteries (MIOCA; ≥50% stenosis, *n* = 237) and MINOCA (<50% stenosis, *n* = 50). In addition, an ischemic control group without obstructive disease (INOCA, *n* = 50) was included for comparative analysis. Laboratory tests, echocardiography, and LV-GLS were assessed at baseline and at 3 months. **Results**: Baseline left ventricular ejection fraction (LVEF) and LV-GLS were significantly lower in MIOCA compared with MINOCA (LVEF: 52.9 ± 10.3% vs. 59.7 ± 8.7%, *p* < 0.001; LV-GLS: −11.46 ± 4.84% vs. −12.63 ± 6.33%, *p* = 0.007, respectively). At 3 months, LVEF remained lower in the MIOCA group (57.5 ± 6.6% vs. 62.0 ± 7.9%; *p* < 0.001, respectively), whereas LV-GLS improved similarly in both groups with no significant difference (−15.27 ± 2.60% vs. −14.87 ± 5.21%; *p* = 0.29, respectively). Among MINOCA patients, the neutrophil-to-platelet ratio (NPR) (*p* = 0.017) and admission glucose (*p* = 0.047) were independently associated with impaired LV-GLS (defined as values greater than −16%). **Conclusions**: Although LVEF remained higher in MINOCA patients during follow-up, LV-GLS impairment persisted at levels comparable to those observed in MIOCA, suggesting ongoing subclinical myocardial dysfunction. LV-GLS offers superior diagnostic sensitivity to LVEF. Inflammatory and glycemic markers may aid in early risk stratification and guide management in patients with MINOCA.

## 1. Introduction

Acute myocardial infarction (MI) remains a leading cause of morbidity and mortality worldwide. In a subset of patients, clinical symptoms and biomarker elevation are consistent with MI, yet coronary angiography reveals no obstructive coronary artery disease. This condition, termed myocardial infarction with non-obstructive coronary arteries (MINOCA), accounts for approximately 6–15% of all MI presentations and represents a clinically challenging and heterogeneous entity [1,2]. The lack of well-defined diagnostic and therapeutic algorithms further contributes to uncertainty in clinical practice [3].

Although left ventricular ejection fraction (LVEF) is often preserved in patients with MINOCA, this does not exclude the presence of subclinical myocardial dysfunction. In recent years, left ventricular global longitudinal strain (LV-GLS), assessed by speckle-tracking echocardiography, has emerged as a more sensitive marker than LVEF for detecting subtle myocardial impairment. LV-GLS enables the earlier identification of minimal myocardial injury and may provide additional diagnostic value across different forms of myocardial infarction, including MINOCA [4].

Recent CMR-based studies have highlighted the value of myocardial strain analysis in MINOCA, demonstrating its ability to detect subtle myocardial dysfunction and differentiate infarction with and without obstructive coronary artery disease [5,6]. These findings support the potential role of myocardial deformation imaging in the evaluation of MINOCA.

The pathophysiology of MINOCA is diverse and may involve mechanisms such as coronary vasospasm, microvascular dysfunction, transient thrombosis, or spontaneous coronary artery dissection (SCAD). Moreover, conditions including Takotsubo cardiomyopathy and myocarditis constitute important differential diagnoses that must be excluded based on clinical and imaging findings. Failure to distinguish between these entities may lead to misinterpretation of the underlying pathology. Consequently, advanced imaging methods capable of identifying not only structural but also functional abnormalities are essential [2,3,7].

Inflammation is known to play a central role in the development and progression of cardiovascular diseases, including acute coronary syndromes. Biomarkers such as the neutrophil-to-lymphocyte ratio (NLR), the neutrophil-to-platelet ratio (NPR), C-reactive protein (CRP), and serum glucose provide accessible measures of systemic inflammation and have demonstrated prognostic value in various cardiovascular conditions [8,9,10,11]. The association of these inflammatory indicators with sensitive functional markers, such as LV-GLS, may be relevant in the assessment of myocardial dysfunction, including in patients with MINOCA.

The present study aimed to evaluate the diagnostic and follow-up value of LV-GLS in patients with MINOCA by comparing strain parameters with those of patients with MIOCA and INOCA, assessing temporal changes over a 3-month follow-up period, and investigating their association with inflammatory and glycemic biomarkers (NLR, NPR, CRP, and glucose).

## 2. Materials and Methods

### 2.1. Study Design and Population

This study included 287 patients who presented to the emergency department of the Istanbul Faculty of Medicine with suspected acute coronary syndrome (ACS) between January and June 2024 and underwent coronary angiography.

Based on coronary angiographic findings and contemporary MINOCA definitions, patients were classified as MIOCA or MINOCA. Patients with at least one coronary artery demonstrating ≥50% stenosis were categorized as having myocardial infarction with obstructive coronary arteries (MIOCA; *n* = 237), whereas patients without obstructive coronary artery disease (no coronary stenosis ≥50%) were classified as having myocardial infarction with non-obstructive coronary arteries (MINOCA; *n* = 50) [12]. Additionally, 50 patients with no significant coronary stenosis but documented myocardial ischemia on non-invasive tests (INOCA) were included as a non-ACS control group.

This study was approved by the Institutional Clinical Research Ethics Committee of Istanbul University, Istanbul Faculty of Medicine (Date: 18 January 2024, Decision No: 2358979).

### 2.2. Inclusion and Exclusion Criteria

The criteria for inclusion included patients aged ≥18 years who were diagnosed with ACS and underwent coronary angiography. The exclusion criteria for the study included patients with myocarditis, cardiomyopathy, inadequate image quality, cardiac amyloidosis, prosthetic valves, significant valvular heart disease, active infection, or morbid obesity and patients aged <18 years.

### 2.3. Clinical and Laboratory Data

Demographic characteristics and cardiovascular risk factors (hypertension, diabetes, hyperlipidemia, smoking, family history) of all patients were recorded. Blood samples were obtained for routine biochemical parameters, including glucose, CRP, lipid profile, HbA1c, troponin, and complete blood count. Inflammatory indices, including the NLR, the NPR, and the platelet/lymphocyte ratio (PLR), were calculated.

### 2.4. Echocardiographic Assessment

All patients underwent transthoracic echocardiography (TTE) using a Siemens Acuson S2000 Prime device (Siemens Healthineers, Erlangen, Germany) with a 1.25–4.5 MHz probe in the left lateral decubitus position. Parasternal long- and short-axis and apical two-, three-, and four-chamber views were obtained. All measurements were performed by a single experienced operator in accordance with the recommendations of the European Association of Cardiovascular Imaging (EACVI) and the American Society of Echocardiography (ASE). The same protocol was repeated during the 3-month follow-up.

Left ventricular ejection fraction (LVEF) was assessed using the biplane Simpson method from apical two- and four-chamber views in accordance with current echocardiographic recommendations. Left ventricular end-diastolic diameter (LVEDD), end-systolic diameter (LVESD), interventricular septal thickness, and posterior wall thickness were assessed using M-mode imaging. Left atrial diameter was measured at end-systole in the parasternal long-axis view.

Left ventricular global longitudinal strain (LV-GLS) was analyzed using Siemens Velocity Vector Imaging (VVI) software from apical two-, three-, and four-chamber images. The frame rate was optimized between 60 and 90 frames per second. At the end-diastole, the apex and basal segments were marked, endocardial borders were automatically tracked, and manual adjustments were applied if necessary. The software divided each wall into three segments, generating 17-segment strain curves; global strain was calculated as the average of all segmental values. Systolic strain values were timed according to aortic valve closure (AVC). Cases with inadequate segment tracking were excluded.

A cut-off value of −16% was used to define impaired LV-GLS. Values greater than −16% (less negative strain values) were considered indicative of impaired myocardial deformation for statistical analyses. Inter-observer reproducibility of LV-GLS measurements was evaluated in a subset of 50 randomly selected patients. Measurements were independently repeated by a second experienced observer, and inter-observer agreement was assessed using the intraclass correlation coefficient (ICC) based on a two-way mixed-effects model with absolute agreement. In addition, Bland–Altman analysis was performed to further evaluate agreement between observers.

### 2.5. Statistical Analysis

The data were analyzed using the Statistical Package for the Social Sciences (SPSS) v27.0 (IBM Corp., Armonk, NY, USA). For continuous variables, the independent samples t-test or analysis of variance (ANOVA) was used for normally distributed data, and the Mann–Whitney U test or the Kruskal–Wallis test was used for non-normally distributed data. Normally distributed variables were expressed as mean ± standard deviation, and non-normally distributed variables were expressed as median (minimum–maximum). Categorical variables were evaluated using the chi-square test. Post hoc pairwise comparisons were performed using Bonferroni correction to adjust for multiple testing. For three-group comparisons, an adjusted significance level of *p* < 0.0167 was considered statistically significant.

Correlations between continuous variables were evaluated using Pearson or Spearman correlation analyses based on distribution characteristics. To assess changes in LVEF and LV-GLS between baseline and month 3, a paired t-test or Wilcoxon signed-rank test was used. A two-way repeated measures ANOVA was performed to examine the interaction between time (baseline vs. month 3) and group (MIOCA vs. MINOCA).

Multivariate logistic regression was performed to identify independent predictors of LV-GLS impairment. For regression analysis, LV-GLS was dichotomized based on a predefined cut-off value of −16%. Values greater than −16% (i.e., less negative strain values) were considered impaired, and logistic regression analysis was performed to identify factors associated with LV-GLS impairment. Variables with a *p*-value < 0.05 in the univariate analysis were entered into the multivariable logistic regression model. A backward stepwise elimination method was used to identify independent predictors. To improve clinical interpretability, the neutrophil-to-platelet ratio (NPR) was rescaled by a factor of 100, and the corresponding odds ratios were expressed per 0.01 increase in NPR. A receiver operating characteristic (ROC) curve analysis was used to determine diagnostic cut-off values and predictive accuracy. A *p*-value < 0.05 was considered statistically significant.

## 3. Results

### 3.1. Demographic and Clinical Characteristics

A total of 337 participants were included in the study, comprising 287 consecutive patients presenting with ACS (237 MIOCA and 50 MINOCA) and an additional 50 patients with INOCA who served as a non-ACS control group. Among the 287 ACS patients, the prevalence of MINOCA was 17.4%.

Of the study population, 71.2% were male (*n* = 240) and 28.8% were female (*n* = 97), with a mean age of 56.9 ± 14 years. The mean age was 60.2 ± 11.6 years in the MIOCA group, 49.9 ± 19 years in the MINOCA group, and 48.7 ± 13.1 years in the control group. Patients in the MIOCA group were significantly older than those in the MINOCA and control groups (*p* < 0.001 for both comparisons). The proportion of males was also significantly higher in the MIOCA group compared with the other two groups (*p* < 0.001).

Diabetes mellitus was identified in 33.3% (*n* = 79) of the MIOCA group, 16% (*n* = 8) of the MINOCA group, and 18% (*n* = 9) of the control group. The prevalence of diabetes was significantly higher in the MIOCA group than in the MINOCA and control groups (*p* = 0.015 and *p* = 0.033, respectively).

A family history of ischemic heart disease was present in 34.6% of the MIOCA group and 24% of the MINOCA group, with no significant difference between the two (*p* = 0.147). However, the rate was notably lower in the control group (1%), which differed significantly from both MI groups (*p* = 0.001 and *p* < 0.001).

Smoking prevalence was markedly higher in the MIOCA group (70%) compared with the MINOCA (34%) and control groups (6%) (*p* < 0.001 for both comparisons). The clinical and demographic features of the groups are presented in Table 1. Additional information regarding ACS presentation, myocardial infarction localization, and management strategy is provided in Supplementary Table S1.

### 3.2. Laboratory Findings

Hemoglobin, platelet count, lymphocyte count, and LDL-cholesterol levels did not differ significantly among the groups. White blood cell count, neutrophil count, NLR, NPR, and CRP levels were significantly higher in both the MINOCA and MIOCA groups than in the control group (*p* < 0.001).

HbA1c and admission glucose levels were significantly higher in the MIOCA group than in the MINOCA group (*p* = 0.006 and *p* < 0.001, respectively). Although LDL cholesterol levels were numerically higher in the MIOCA group, the difference was not statistically significant (*p* = 0.09).

Admission hs-troponin I levels were borderline significantly higher in the MINOCA group compared with the MIOCA group (*p* = 0.039). Peak hs-troponin I levels, however, were significantly higher in the MIOCA group than in the MINOCA group (*p* < 0.001).

ProBNP levels were significantly higher in both MI groups (MINOCA and MIOCA) compared with the control group, while no significant difference was observed between the two MI groups (*p* > 0.05). The laboratory findings of the groups are presented in Table 2.

### 3.3. Echocardiographic Findings

At admission, LVEF and LV-GLS values were significantly lower in the MIOCA group than in the MINOCA group (EF: 52.9 ± 10.1 vs. 59.3 ± 8.5; *p* < 0.001; GLS: −11.5 ± 5 vs. −12.6 ± 6; *p* = 0.007). At the 3-month follow-up, both groups demonstrated notable improvement in EF and LV-GLS. The difference in EF between groups persisted during follow-up (*p* < 0.001), whereas LV-GLS values became similar between the MIOCA and MINOCA groups (−15.3 ± 2 vs. −14.9 ± 5; *p* = 0.29).

**Figure 1:**
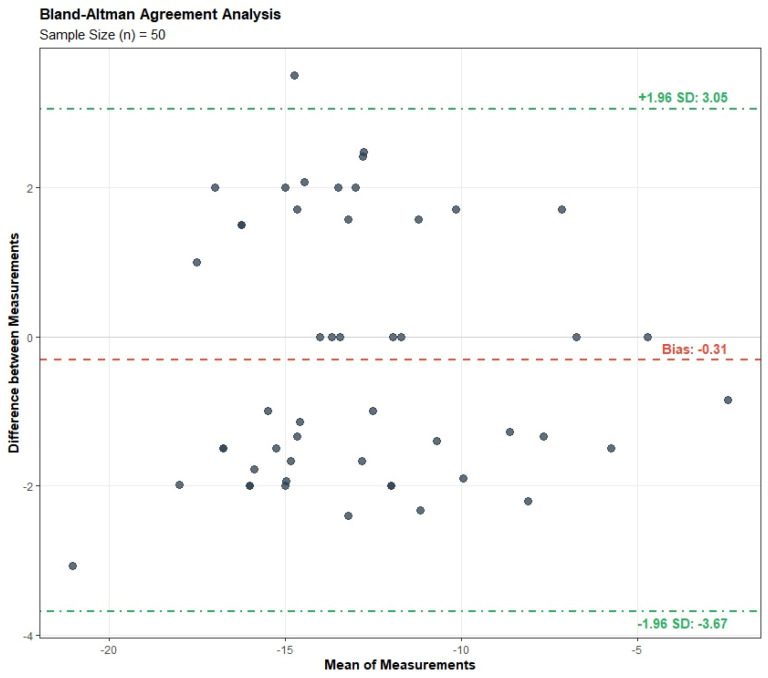
Bland–Altman Plot Demonstrating Inter-Observer Agreement for LV-GLS Measurements. The central dashed line indicates the mean bias, while the upper and lower dashed lines represent the 95% limits of agreement.

Inter-observer reproducibility analysis demonstrated good agreement for LV-GLS measurements, with an ICC of 0.895 (95% CI: 0.822–0.939; *p* < 0.001). The Bland–Altman plot demonstrating inter-observer agreement is presented in Figure 1.

### 3.4. Correlation and Regression Analysis

Baseline LV-GLS was positively correlated with BMI (r = 0.432, *p* = 0.002), the NLR (r = 0.529, *p* < 0.001), the NPR (r = 0.574, *p* < 0.001), admission glucose (r = 0.357, *p* = 0.012), CRP (r = 0.303, *p* = 0.033), proBNP (r = 0.587, *p* < 0.001), and HbA1c (r = 0.314, *p* = 0.030). No correlation was observed with admission or peak troponin levels.

At the 3-month follow-up, LV-GLS remained positively correlated with BMI (r = 0.335, *p* = 0.017), the NLR (r = 0.343, *p* = 0.015), the NPR (r = 0.295, *p* = 0.037), admission glucose (r = 0.336, *p* = 0.018), and proBNP (r = 0.493, *p* < 0.001). No correlation was found between follow-up LV-GLS and CRP or HbA1c. Correlation analyses of baseline and follow-up LV-GLS in the MINOCA group are presented in Table 3.

Baseline LV-GLS in the MIOCA group was positively correlated with the NLR (r = 0.532, *p* < 0.001), the NPR (r = 0.350, *p* = 0.004), the PLR (r = 0.456, *p* < 0.001), admission glucose (r = 0.509, *p* < 0.001), CRP (r = 0.585, *p* < 0.001), and proBNP (r = 0.452, *p* < 0.001). No significant correlation was observed with age, BMI, creatinine, admission troponin, peak troponin, or HbA1c.

At the 3-month follow-up, LV-GLS remained positively correlated with the NLR (r = 0.363, *p* = 0.029), the PLR (r = 0.402, *p* = 0.015), admission glucose (r = 0.528, *p* = 0.001), and CRP (r = 0.410, *p* = 0.013). No significant correlation was found between follow-up LV-GLS and age, BMI, the NPR, creatinine, proBNP, admission troponin, peak troponin, or HbA1c.

Correlation analyses of baseline and follow-up LV-GLS in the MIOCA group are presented in Table 4.

Independent predictors of LV-GLS impairment were evaluated using univariate and multivariate logistic regression analyses. Variables found to be significant in univariate analysis and clinically relevant parameters were included in the multivariate model. In the final multivariate model, the NPR (per 0.01 increase; OR 1.18, *p* = 0.017) and admission glucose (OR 1.051, *p* = 0.047) were identified as independent predictors of LV-GLS impairment in MINOCA patients. Other variables lost significance in the multivariate analysis. Results of the univariate and multivariate logistic regression analyses are presented in Table 5.

**Figure 2:**
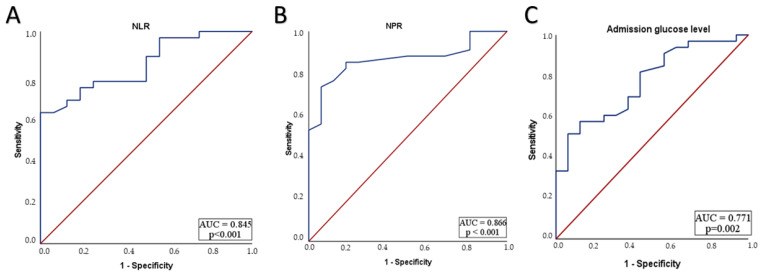
ROC Curves of the NLR, the NPR, and Admission Glucose for Predicting LV-GLS Impairment in Patients with MINOCA. (**A**) The neutrophil-to-lymphocyte ratio (NLR), (**B**) the neutrophil-to-platelet ratio (NPR), and (**C**) admission glucose level for predicting impaired LV-GLS in patients with MINOCA. The red dashed line indicates the reference line.

ROC analysis identified admission glucose, the NLR, and the NPR as significant predictors of LV-GLS impairment. Detailed ROC analysis results, including AUC values, cut-off points, sensitivity, and specificity, are summarized in Table 6, while the ROC curves are presented in Figure 2A–C.

## 4. Discussion

This study suggests that strain echocardiography (LV-GLS) may provide additional diagnostic value beyond conventional ejection fraction assessment in evaluating left ventricular function among patients with MINOCA.

The significant correlations observed between LV-GLS and admission glucose levels, as well as inflammatory markers such as the NLR and NPR, suggest that myocardial dysfunction may be influenced not only by anatomical factors but also by systemic metabolic and inflammatory processes across different forms of myocardial infarction, including MINOCA.

The heterogeneous pathophysiology of MINOCA has been emphasized in previous studies [7,13]. Approximately half of the cases are attributed to atherosclerotic mechanisms, such as plaque rupture or erosion, while the remainder are related to non-atherosclerotic causes, including coronary vasospasm, microvascular dysfunction, or thromboembolism [3,12,14]. In this study, the younger age and higher proportion of women in the MINOCA group supported this distinct pathophysiological profile.

However, the similar prevalence of hypertension, hyperlipidemia, and family history of ischemic heart disease between the MINOCA and MIOCA groups indicates that shared atherosclerotic risk factors may still play a contributory role in both conditions. The markedly higher rates of diabetes and smoking in the MIOCA group, on the other hand, suggest that these two factors may exert a stronger influence on the development of obstructive coronary disease.

In our cohort, classical inflammatory markers were comparably elevated in both the MINOCA and MIOCA patients. This finding supports the notion that inflammation plays a key role in acute vascular events, even in the absence of obstructive coronary lesions. The significantly lower values observed in the control group further imply that inflammation is strongly associated with the ACS process.

Hematologic biomarkers, such as the NLR, the PLR, and the NPR, were markedly higher in both MI groups, indicating that these indices may reflect not only the degree of systemic inflammation but also the presence of subclinical myocardial injury. Previous studies have shown associations between these markers and increased mortality and complication rates, and our findings are consistent with this literature [9,10,11,15,16,17,18].

Although troponin levels were significantly higher in the MIOCA group, proBNP levels were comparable between the two MI groups. This pattern suggests that myocardial dysfunction in MINOCA may involve more subtle or persistent impairment, despite relatively limited myocardial injury [19,20]. The prognostic value of proBNP in this context is also consistent with earlier studies [21].

In this study, both EF and GLS values at admission were significantly lower in the MIOCA group compared with the MINOCA group, a finding consistent with more extensive myocardial injury associated with obstructive coronary lesions. Although EF and LV-GLS values improved in both groups at the 3-month follow-up, the degree of GLS improvement was more pronounced in the MIOCA group. Ultimately, the GLS values became comparable between the groups at follow-up, suggesting that subclinical left ventricular dysfunction may persist in MINOCA despite the absence of obstructive coronary disease, highlighting the potential role of LV-GLS in detecting subtle myocardial impairment.

Multiple studies have demonstrated that LV-GLS provides a more sensitive assessment of subclinical LV dysfunction than EF [22,23]. Strain parameters have also been shown to correlate with the severity and extent of coronary artery disease. For example, Goswami et al. reported a significant association between LV-GLS and SYNTAX scores in patients with non-ST-segment elevation myocardial infarction (NSTEMI), highlighting its ability to distinguish between left main or triple-vessel disease [22]. Similarly, İnci et al. identified subclinical myocardial dysfunction using LV-GLS in patients with MINOCA, underscoring its diagnostic utility [24]. Furthermore, Shen et al. demonstrated that myocardial deformation parameters derived from cardiac magnetic resonance imaging could differentiate myocardial infarction with and without obstructive coronary artery disease, further supporting the value of strain assessment in MINOCA [6]. Our results support the use of strain analysis as a more sensitive tool for detecting subtle functional abnormalities in MINOCA.

The more pronounced treatment response observed in the MIOCA group suggests that ventricular dysfunction in MINOCA may be related to chronic, less reversible processes. However, the more pronounced improvement in LV-GLS observed in the MIOCA group may also be influenced by differences in therapeutic strategies. Patients with MIOCA are more likely to undergo revascularization procedures and receive guideline-directed medical therapy, which are known to promote myocardial recovery and reverse remodeling. In contrast, patients with MINOCA often have more heterogeneous management approaches. Therefore, the observed differences in LV-GLS recovery between the groups may not solely reflect underlying pathophysiological mechanisms but could also be partially explained by treatment-related factors.

Non-atherosclerotic mechanisms, such as microvascular dysfunction, vasospasm, or spontaneous coronary artery dissection—conditions more frequently implicated in MINOCA—may contribute to limited recovery, especially given the lack of well-defined treatment strategies targeting these pathways.

Strain echocardiography has also been shown to predict future cardiovascular events. Lenell et al. demonstrated that LV-GLS provides valuable prognostic information beyond EF in long-term risk assessments [25]. Therefore, LV-GLS emerges not only as a diagnostic parameter but also as a clinically relevant prognostic tool.

In our analysis, significant correlations were observed between LV-GLS and inflammatory (NLR, NPR, CRP, neutrophil count) and glycemic (glucose) markers in both MINOCA and MIOCA patients. However, in the context of MINOCA—where overt obstructive coronary lesions are absent—these associations may reflect a more prominent role of systemic inflammatory and metabolic processes in the development of subclinical myocardial dysfunction.

Markers such as the NLR, the NPR, and admission glucose were associated with LV-GLS impairment and may have potential diagnostic and prognostic relevance. Previous studies have shown that the NLR is associated with acute myocardial injury and short-term mortality in ST-segment elevation myocardial infarction (STEMI) patients [15,16]. Similarly, NPR has been identified as a prognostic indicator in both obstructive and non-obstructive presentations [9].

Glucose levels were higher in both the MINOCA and MIOCA groups compared with controls, and their association with LV-GLS reinforced the impact of hyperglycemia on myocardial dysfunction. Studies by Paolisso et al. demonstrated the differential physiological effects of hyperglycemia in MINOCA and MIOCA, emphasizing its influence on inflammatory activation and myocardial healing [26,27]. Additionally, Sia et al. showed that admission glucose and stress hyperglycemia ratios predict one-year mortality in AMI patients, underscoring the importance of integrating these markers into clinical decision making [28]. Our findings similarly highlight the potential prognostic importance of early glycemic assessment in MINOCA.

In summary, this study demonstrated that strain echocardiography provides meaningful diagnostic insight into left ventricular dysfunction in MINOCA. LV-GLS proves to be more sensitive than EF in detecting subclinical myocardial impairment. The associations between LV-GLS and inflammatory and glycemic markers suggest that myocardial dysfunction in MINOCA reflects not only anatomical but also systemic processes. The limited improvement in GLS at 3 months highlights the potentially chronic or less reversible nature of dysfunction in MINOCA. Combined assessment using LV-GLS and readily available biomarkers may therefore support earlier diagnosis and more targeted clinical management strategies in this heterogeneous patient population.

## 5. Limitations

This study has several limitations. First, due to its prospective observational design, causal relationships cannot be definitively established. Second, the relatively small sample size, particularly in the MINOCA subgroup, may limit the generalizability of the findings.

Third, advanced diagnostic modalities were not systematically performed in all patients. Cardiac magnetic resonance imaging (CMR) was selectively used only in cases with a high clinical suspicion of myocarditis, and patients with confirmed myocarditis were excluded. However, the absence of routine CMR, as well as the lack of intravascular imaging techniques such as optical coherence tomography (OCT), vasospasm provocation testing, and physiological assessment of intermediate coronary lesions (e.g., FFR), limited our ability to fully elucidate the underlying mechanisms of MINOCA. Consequently, some patients classified as MINOCA may have had unrecognized non-ischemic myocardial injury, including myocarditis or Takotsubo syndrome, which may have influenced the observed findings.

Finally, detailed therapeutic strategies and long-term prognostic outcomes were not systematically evaluated, which may restrict the interpretation of the clinical implications of our findings. In addition, the relatively small MINOCA sample size may have limited the stability of the multivariable regression analyses, and the ROC-derived cut-off values were not externally validated. Therefore, these findings should be considered exploratory and hypothesis-generating. In addition, absolute LV-GLS values may be influenced by vendor-specific software and strain analysis algorithms, which may limit direct comparison of our strain measurements with those reported in studies using different echocardiographic platforms. Future studies with larger sample sizes, comprehensive imaging approaches, and long-term follow-up are needed to validate and extend these results.

## 6. Conclusions

This study suggests that strain echocardiography may be a sensitive tool for detecting subclinical left ventricular dysfunction in patients presenting with acute coronary syndromes, including those with MINOCA, despite preserved LVEF. The associations observed between LV-GLS and glycemic, as well as inflammatory markers, suggest that myocardial dysfunction in ACS may be influenced not only by anatomical factors but also by systemic metabolic and inflammatory processes. These mechanisms are not specific to MINOCA; however, their presence in MINOCA patients highlights the potential contribution of non-obstructive pathophysiological pathways in this subgroup. The limited improvement in LV-GLS during follow-up suggests that persistent subclinical myocardial dysfunction may remain clinically relevant in MINOCA and warrants further investigation. Overall, incorporating LV-GLS assessment together with inflammatory and glycemic markers may provide additional insight for early functional evaluation and risk stratification in patients with MINOCA.

## Figures and Tables

**Table 1:** Baseline demographic and clinical characteristics of the study population categorized by group.

Variables	MINOCA (*n* = 50)	MIOCA (*n* = 237)	INOCA (*n* = 50)	*p*-Value
Age (years)	49.86 ± 19.01 ^c^	60.16 ± 11.61 ^b,c^	48.68 ± 13.06 ^b^	<0.001
Male sex (*n*, %)	27 (%54) ^c^	189 (%79.7) ^b,c^	24 (%48) ^b^	<0.001
Body mass index (kg/m^2^)	27.66 ± 5.14	27.7 ± 4.35	26.69 ± 4.58	0.34
Hypertension (*n*, %)	23 (%46)	110 (%46.4)	16 (%32)	0.169
Diabetes mellitus (*n*, %)	8 (%16) ^c^	79 (%33.3) ^c^	9 (%18)	0.010
Chronic kidney disease (*n*, %)	0 (%0)	15 (%6.3)	0 (%0)	0.036
Family history	12 (%24) ^a^	82 (%34.6) ^b^	1 (%2) ^a,b^	<0.001
Smoking (*n*, %)	17 (%34) ^a,c^	166 (%70) ^b,c^	3 (%6) ^a,b^	<0.001

Post hoc pairwise comparisons were performed with Bonferroni correction. ^a^—Statistically significant difference between INOCA and MINOCA; ^b^—Statistically significant difference between INOCA and MIOCA; ^c^—Statistically significant difference between MINOCA and MIOCA.

**Table 2:** Comparison of laboratory and echocardiographic parameters among the MINOCA, MIOCA, and INOCA groups.

Variables	MINOCA (*n* = 50)	MIOCA (*n* = 237)	INOCA (*n* = 50)	*p*-Value
White blood cell (10^3^/μL)	9.36 ± 0.5 ^a^	10.68 ± 0.52 ^b^	6.67 ± 0.22 ^a,b^	<0.001
Hemoglobin, (10^6^/μL)	13.75 ± 0.23	13.64 ± 0.12	13.54 ± 0.19	0.637
Platelet, (10^3^/μL)	240.52 ± 11.15	245.41 ± 4.62	237.37 ± 6.73	0.807
Neutrophil, (10^3^/μL)	6.28 ± 0.46 ^a^	6.86 ± 0.18 ^b^	3.96 ± 0.19 ^a,b^	<0.001
Lymphocyte, (10^3^/μL)	2.13 ± 0.13	2.39 ± 0.09	2.11 ± 0.07	0.652
Neutrophil-to-lymphocyte ratio	3.85 ± 0.55 ^a^	3.95 ± 0.21 ^b^	1.92 ± 0.08 ^a,b^	<0.001
Neutrophil-to-platelet ratio	0.028 ± 0.002 ^a^	0.030 ± 0.001 ^b^	0.017 ± 0.001 ^a,b^	<0.001
Platelet-to-lymphocyte ratio	128.9 ± 8.9	133.3 ± 6.1	117.8 ± 4.8	0.620
Admission glucose level, (mg/dL)	124.78 ± 40.5 ^a,c^	164.86 ± 88.5 ^b,c^	97.54 ± 24 ^a,b^	<0.001
Serum creatinine, (mg/dL)	0.86 ± 0.18 ^c^	1.05 ± 0.8 ^b,c^	0.79 ± 0.15 ^b^	<0.001
C-reactive protein, (mg/L)	30.54 ± 48.9 ^a^	16.24 ± 26.7 ^b^	3.48 ± 4.8 ^a,b^	<0.001
Pro-B-type natriuretic peptide, (pg/mL)	1371.41 ± 2417 ^a^	1669.78 ± 4025 ^b^	69.02 ± 50 ^a,b^	<0.001
Admission high-sensitivity troponin, (pg/mL)	440.64 ± 875 ^a^	428.24 ± 1753 ^b^	4.20 ± 4.1 ^a,b^	<0.001
Peak high-sensitivity troponin, (pg/mL)	840.26 ± 1176	10,427.51 ± 15,489		<0.001
HbA1c, (%)	5.84 ± 0.85 ^c^	6.59 ± 1.81 ^b,c^	5.52 ± 0.76 ^b^	<0.001
Left ventricular global longitudinal strain, (%)	−12.63 ± 6.33 ^a,c^	−11.46 ± 4.84 ^b,c^	−19.68 ± 2.18 ^a,b^	<0.001
Left ventricular ejection fraction, (%)	59.26 ± 8.46 ^a,c^	52.93 ± 10.12 ^b,c^	64.27 ± 3.95 ^a,b^	<0.001
LV-GLS at 3-month follow-up, (%)	−14.87 ± 5.21	−15.27 ± 2.6		0.29
LVEF at 3-month follow-up, (%)	61.96 ± 7.84	57.49 ± 6.47		<0.001

Post hoc pairwise comparisons were performed with Bonferroni correction. ^a^—Statistically significant difference between INOCA and MINOCA; ^b^—Statistically significant difference between INOCA and MIOCA; ^c^—Statistically significant difference between MINOCA and MIOCA.

**Table 3:** Correlation of LV-GLS with inflammatory, metabolic, and cardiac biomarkers in the MINOCA group at baseline and follow-up.

Variables	LV-GLS		LV-GLS at 3-Month Follow-Up	
	rho	*p*-Value	rho	*p*-Value
Age	0.209	0.145	0.161	0.265
Body mass index	0.432	0.002	0.335	0.017
Neutrophil-to-lymphocyte ratio	0.529	<0.001	0.343	0.015
Neutrophil-to-platelet ratio	0.574	<0.001	0.295	0.037
Platelet-to-lymphocyte ratio	0.161	0.263	0.200	0.165
Admission glucose level	0.357	0.012	0.336	0.018
Serum creatinine	0.132	0.360	0.154	0.287
C-reactive protein	0.303	0.033	0.141	0.329
Pro-B-type natriuretic peptide	0.587	<0.001	0.493	<0.001
Admission high-sensitivity troponin	0.093	0.521	−0.090	0.536
Peak high-sensitivity troponin	0.053	0.713	−0.090	0.534
HbA1c	0.314	0.030	0.278	0.056

**Table 4:** Correlation of LV-GLS with inflammatory, metabolic, and cardiac biomarkers in the MIOCA group at baseline and follow-up.

Variables	LV-GLS		LV-GLS at 3-Month Follow-Up	
	rho	*p*-Value	rho	*p*-Value
Age	0.184	0.142	0.077	0.656
Body mass index	0.072	0.571	0.057	0.743
Neutrophil-to-lymphocyte ratio	0.532	<0.001	0.363	0.029
Neutrophil-to-platelet ratio	0.350	0.004	0.248	0.145
Platelet-to-lymphocyte ratio	0.456	<0.001	0.402	0.015
Admission glucose level	0.509	<0.001	0.528	0.001
Serum creatinine	−0.038	0.762	−0.052	0.765
C-reactive protein	0.585	<0.001	0.41	0.013
Pro-B-type natriuretic peptide	0.452	<0.001	0.166	0.342
Admission high-sensitivity troponin	0.175	0.167	−0.115	0.512
Peak high-sensitivity troponin	0.230	0.067	0.217	0.210
HbA1c	0.179	0.161	0.042	0.811

**Table 5:** Logistic regression analyses for determinants of LV-GLS impairment in MINOCA.

Variables	Univariate Regression			Multivariate Regression		
	OR	95% CI	*p*-Value	OR	95% CI	*p*-Value
Age	1.023	0.99–1.06	0.173			
Male sex	0.404	0.12–1.42	0.156			
Body mass index	1.222	1.03–1.45	0.021	1.32	0.97–1.8	0.073
Hemoglobin	0.906	0.63–1.31	0.599			
Neutrophil-to-lymphocyte ratio	3.934	1.48–10.46	0.006	1.95	0.56–6.8	0.296
Neutrophil-to-platelet ratio (per 0.01 increase)	1.269	1.083–1.487	0.001	1.18	1.02–1.372	0.017
Platelet-to-lymphocyte ratio	1.007	0.995–1.02	0.26			
Admission glucose level	1.041	1.009–1.07	0.011	1.051	1.001–1.103	0.047
C-reactive protein	1.016	0.996–1.04	0.120			
Pro-B-type natriuretic peptide	1.001	1.000–1.003	0.079			
Admission high-sensitivity troponin	1.000	0.999–1.001	0.710			
Peak high-sensitivity troponin	1.000	0.999–1	0.832			
HbA1c	2.936	1.03–8.4	0.045	1.61	0.16–16	0.68

NPR odds ratios are expressed per 0.01 increase in the NPR.

**Table 6:** ROC analysis of biomarkers for predicting LV-GLS impairment in MINOCA.

Variable	AUC, (95% CI)	Cut-Off	Sensitivity (%)	Specificity (%)	*p* Value
NLR	0.845, (0.740–0.951)	2.23	77	75	<0.001
NPR	0.866, (0.764–0.967	0.02	85	81	<0.001
Glucose	0.771, (0.637–0.905)	108	61	75	0.002

## Data Availability

The data presented in this study are available on request from the corresponding author. The data are not publicly available due to ethical reasons.

## Supplementary Materials

The following supporting information can be downloaded at https://www.mdpi.com/article/10.3390/jcm15134934/s1. Table S1: Clinical presentation and management strategy of patients with MIOCA and MINOCA.

## Abbreviations

ACS	Acute coronary syndrome
AMI	Acute myocardial infarction
ANOVA	Analysis of variance
ASE	American Society of Echocardiography
AVC	Aortic valve closure
BMI	Body mass index
CRP	C-reactive protein
EACVI	European Association of Cardiovascular Imaging
EF	Ejection fraction
HbA1c	Hemoglobin A1c
hs-Troponin I	High-sensitivity troponin I
INOCA	Ischemia with non-obstructive coronary arteries
LDL	Low-density lipoprotein
LV	Left ventricle/Left ventricular
LVEDD	Left ventricular end-diastolic diameter
LVEF	Left ventricular ejection fraction
LVESD	Left ventricular end-systolic diameter
LV-GLS	Left ventricular global longitudinal strain
MI	Myocardial infarction
MINOCA	Myocardial infarction with non-obstructive coronary arteries
MIOCA	Myocardial infarction with obstructive coronary arteries
NLR	Neutrophil-to-lymphocyte ratio
NPR	Neutrophil-to-platelet ratio
NSTEMI	Non-ST-segment elevation myocardial infarction
PLR	Platelet-to-lymphocyte ratio
proBNP	Pro-B-type natriuretic peptide
ROC	Receiver operating characteristic
SCAD	Spontaneous coronary artery dissection
SPSS	Statistical Package for the Social Sciences
STEMI	ST-segment elevation myocardial infarction
TTE	Transthoracic echocardiography
VVI	Velocity Vector Imaging
