# Supplementary material for: Strain Echocardiography in MINOCA: Diagnostic and Follow-Up Implications
Source: J Clin Med. 2026 Jun 25;15(13):4934. doi: 10.3390/jcm15134934 (PMC13361653; doi:10.3390/jcm15134934)
Supplement: Supplementary file 1 [file jcm-15-04934-s001.zip › jcm-4365950-supplementary.pdf]

Supplementary Table S1. Clinical presentation and management strategy of patients with MIOCA and MINOCA

Clinical Category	Total n (%)	PCI n	Medical Management n
**MIOCA**	237 (82.6%)	198	39
— NSTEMI	120 (41.8%)	81	39
— STEMI – Anterior	53 (18.5%)	53	0
— STEMI – Inferior	64 (22.3%)	64	0
**MINOCA**	50 (17.4%)	0	50
